# Bounds for the Z-spectral radius of nonnegative tensors

**DOI:** 10.1186/s40064-016-3338-3

**Published:** 2016-10-06

**Authors:** Jun He, Yan-Min Liu, Hua Ke, Jun-Kang Tian, Xiang Li

**Affiliations:** School of Mathematics, Zunyi Normal College, Zunyi, 563002 Guizhou People’s Republic of China

**Keywords:** Bound, Nonnegative tensor, Z-eigenvalue, 15A18, 15A69, 65F15, 65F10

## Abstract

In this paper, we have proposed some new upper bounds for the largest Z-eigenvalue of an irreducible weakly symmetric and nonnegative tensor, which improve the known upper bounds obtained in Chang et al. (Linear Algebra Appl 438:4166–4182, 2013), Song and Qi (SIAM J Matrix Anal Appl 34:1581–1595, 2013), He and Huang (Appl Math Lett 38:110–114, 2014), Li et al. (J Comput Anal Appl 483:182–199, 2015), He (J Comput Anal Appl 20:1290–1301, 2016).

## Background

Let ${\mathbb{R}}$ be the real field. An *m*th order *n* dimensional square tensor $\mathcal {A}$ consists of $n^m$ entries in ${\mathbb{R}}$, which is defined as follows:

$$\begin{aligned} \mathcal {A} = (a_{i_1 i_2 \ldots i_m } ) ,\quad a_{i_1 i_2 \ldots i_m } \in {\mathbb{R}},\quad 1 \le i_1 ,i_2 , \ldots i_m \le n. \end{aligned}$$

$\mathcal {A}$ is called nonnegative if $a_{i_1 i_2 \ldots i_m } \ge 0$. To an n-vector *x*, real or complex, we define the n-vector:

$$\begin{aligned} \mathcal {A}x^{m-1}=\left( \sum \limits _{i_2 , \ldots ,i_m = 1}^n {a_{ii_2 \ldots i_m } }x_{i_2} \ldots x_{i_m}\right) _{1\le i \le n} \end{aligned}$$

and

$$\begin{aligned} x^{[m-1]}= (x_i^{m-1})_{1\le i \le n}. \end{aligned}$$

If $\mathcal {A}x^{m-1}=\lambda x^{[m-1]}$, *x* and $\lambda$ are all real, then $\lambda$ is called an H-eigenvalue of $\mathcal {A}$ and *x* an H-eigenvector of $\mathcal {A}$ associated with $\lambda$. If $\mathcal {A}x^{m-1}=\lambda x$ with $x^Tx=1$, *x* and $\lambda$ are all real, then $\lambda$ is called a Z-eigenvalue of $\mathcal {A}$ and *x* a Z-eigenvector of $\mathcal {A}$ associated with $\lambda$ Qi (2005), Lim (2005). See more about the eigenvalue problems of tensors in Chang et al. (2009, 2010), Qi (2007), Yang and Yang (2010, 2011), Ng et al. (2009), Zhou et al. (2013), Li et al. (2014, 2015), Hu and Huang (2012), Hu et al. (2013).

The following definition for irreducibility has been introduced in Chang et al. (2008) and Lim (2005).

### **Definition 1**

The square tensor $\mathcal {A}$ is called reducible if there exists a nonempty proper index subset ${\mathbb{J}} \subset \{ 1,2,\ldots ,n \}$ such that $a_{i_1,i_2,\ldots ,i_m} = 0,\ \ \forall i_1 \in {\mathbb{J}},\ \ \forall i_2, \ldots , i_m \notin {\mathbb{J}}.$ If $\mathcal {A}$ is not reducible, then we call $\mathcal {A}$ to be irreducible.

### **Definition 2**

Let $\mathcal {A}$ be an m-order and n-dimensional tensor. We define $\sigma (\mathcal {A})$ the Z-spectrum of $\mathcal {A}$ by the set of all Z-eigenvalues of $\mathcal {A}$. Assume $\sigma (\mathcal {A})\ne \emptyset$, then the Z-spectral radius of $\mathcal {A}$ is denoted by

$$\begin{aligned} \rho (\mathcal {A})= \max \{|\lambda |: \lambda \in \sigma (\mathcal {A}) \}. \end{aligned}$$

Let $N=\{1,2, \ldots ,n\}$. In 2013, Chang et al. gave the following bound for the Z-eigenvalues of an *m*-order *n*-dimensional tensor $\mathcal {A}$.

### **Theorem 1**

*Let* $\mathcal {A}$ *be an m-order and n-dimensional tensor. Then*

1 $$\begin{aligned} \rho (\mathcal {A}) \le \sqrt{n} \max _{i\in N} \sum \limits _{i_2 , \ldots ,i_m = 1}^n {\left| {a_{ii_2 \ldots i_m } } \right| }. \end{aligned}$$

For the positively homogeneous operators, Song and Qi (2013) studied the relationship between the Gelfand formula and the spectral radius as well as the upper bound of the spectral radius. From Corollary  4.5 in Song and Qi (2013), we can get the following result:

### **Theorem 2**

*Let* $\mathcal {A}$ *be an m-order and n-dimensional tensor. Then*

2 $$\begin{aligned} \rho (\mathcal {A}) \le \max _{i\in N} \sum \limits _{i_2 , \ldots ,i_m = 1}^n {\left| {a_{ii_2 \ldots i_m } } \right| }. \end{aligned}$$

We shall denote the set of all *m*th order *n* dimensional tensors by ${\mathbb{R}}^{[m,n]}$, and the set of all nonnegative (or, respectively, positive) *m*th order *n* dimensional tensors by ${\mathbb{R}}_+^{[m,n]}$ (or, respectively, ${\mathbb{R}}_{++}^{[m,n]}$). If the tensor is positive, He and Huang gave the following Z-eigenpair bound (see Theorem 2.7 of He and Huang 2014):

### **Theorem 3**

*Suppose that* $\mathcal {A}=(a_{i_1i_2 \ldots i_m}) \in {\mathbb{R}}_{++}^{[m,n]}$ *is an irreducible weakly symmetric tensor. Then*

3 $$\begin{aligned} \rho (\mathcal {A}) \le R-l(1-\theta ), \end{aligned}$$

*where* $$R_i = \sum\nolimits _{i_2 , \ldots ,i_m = 1}^n {|a_{ii_2 \ldots i_m } |}$$,

$$\begin{aligned} R = \mathop {\max }\limits _{i\in N} R_i ,\; r = \mathop {\min }\limits _{i\in N} R_i,\quad l = \mathop {\min }\limits _{i_1 , \ldots ,i_m } a_{i_1 \ldots i_m }, \quad \theta =\left\{ {\frac{{r }}{{R}}} \right\} ^{\frac{1}{{m}}}. \end{aligned}$$

Li et al. obtained the following upper bound (see Theorem 3.5 of Li et al. 2015):

### **Theorem 4**

*Suppose that* $\mathcal {A}=(a_{i_1i_2 \ldots i_m}) \in {\mathbb{R}}_+^{[m,n]}$ *is an irreducible weakly symmetric tensor. Then*

4 $$\begin{aligned} \rho (\mathcal {A}) \le \max _{i,j} \left\{ r_i+a_{ij\ldots j}(\delta ^{-\frac{m-1}{m}}-1) \right\} , \end{aligned}$$

*where*

$$\begin{aligned} \delta = \frac{\min _{i,j}a_{ij \ldots j}}{r-\min _{i,j}a_{ij \ldots j}}\left( \gamma ^{\frac{m-1}{m}}-\gamma ^{\frac{1}{m}}\right) +\gamma , \quad \gamma =\frac{R-\min _{i,j}a_{ij \ldots j}}{r-\min _{i,j}a_{ij \ldots j}}. \end{aligned}$$

A real tensor of order *m* dimension *n* is called the unit tensor, if its entries are $\delta _{i_1\ldots i_m}$ for $i_1,\ldots ,i_m\in N$, where

$$\begin{aligned} \delta _{i_1 \ldots i_m } = \left\{ \begin{array}{lll} 1,&{} \quad \text {if}\; i_1 = \cdots = i_m \\ 0, &{} \quad \text {otherwise}. \\ \end{array} \right. \end{aligned}$$

And we define

$$\begin{aligned} r_i(\mathcal {A})=\sum \limits _{\delta _{ii_2\ldots i_m}= 0} |a_{ii_2\ldots i_m}|, \ \ r_i^j(\mathcal {A})= \mathop{\mathop{\sum} \limits _{\delta _{ii_2\ldots i_m}= 0,}}\limits_ {\delta _{ji_2\ldots i_m}= 0} |a_{ii_2\ldots i_m}|=r_i(\mathcal {A})-|a_{ij\ldots j}|. \end{aligned}$$

He gave the following upper bound (see Theorem 3.3 of He 2016):

### **Theorem 5**

*Suppose that* $\mathcal {A}=(a_{i_1i_2 \ldots i_m}) \in {\mathbb{R}}_+^{[m,n]}$ *is an irreducible weakly symmetric tensor. Then*

5 $$\begin{aligned} \rho (\mathcal {A}) \le \max \limits _{i,j\in N,j\ne i}\frac{1}{2}\left\{ a_{i\ldots i}+a_{j\ldots j}+r_i^j(\mathcal {A})+\Theta _{i,j}^{\frac{1}{2}}(\mathcal {A})\right\} , \end{aligned}$$

*where*

$$\begin{aligned} \Theta _{i,j}(\mathcal {A})=\left( a_{i\ldots i}-a_{j\ldots j}+r_i^j(\mathcal {A})\right) ^2 + 4a_{ij\ldots j}r_j(\mathcal {A}). \end{aligned}$$

Our goal in this paper is to show some tighter upper bounds for the largest Z-eigenvalue of a nonnegative tensor. In section “Main results”, some new upper bounds for the largest Z-eigenvalue are obtained, which are tighter than the results in Theorems 1–5 (Chang et al. 2013; Song and Qi 2013; He and Huang 2014; Li et al. 2015; He 2016).

## Main results

In this section, we consider some new upper bounds for the largest Z-eigenvalue of a nonnegative tensor.

A tensor $\mathcal {A}$ is called *weakly symmetric* if the associated homogeneous polynomial $\mathcal {A}x^m$ satisfies

$$\begin{aligned} \nabla \mathcal {A}x^m=m\mathcal {A}x^{m-1}. \end{aligned}$$

This concept was first introduced and used by Chang et al. (2013) for studying the properties of *Z*-eigenvalue of nonnegative tensors and presented the following Perron-Frobenius Theorem for the *Z*-eigenvalue of nonnegative tensors.

### **Lemma 1**

*Suppose that* $\mathcal {A}=(a_{i_1i_2 \ldots i_m}) \in {\mathbb{R}}_+^{[m,n]}$ *is an irreducible weakly symmetric tensor, then the spectral radius* $\rho (\mathcal {A})$ *is a positive Z-eigenvalue with a positive Z-eigenvector.*

Based on the lemma, we give our main results as follows.

### **Theorem 6**

*Suppose that* $\mathcal {A}=(a_{i_1i_2 \ldots i_m}) \in {\mathbb{R}}_+^{[m,n]}$ *is an irreducible weakly symmetric tensor. Then*

$$\begin{aligned} \rho (\mathcal {A}) \le \max \limits _{i\in N} \min \limits _{j\in N,j\ne i}\frac{1}{2}\left\{ a_{i\ldots i}+a_{j\ldots j}+r_i^j(\mathcal {A})+\Theta _{i,j}^{\frac{1}{2}}(\mathcal {A})\right\} , \end{aligned}$$

*where*

$$\begin{aligned} \Theta _{i,j}(\mathcal {A})=\left( a_{i\ldots i}-a_{j\ldots j}+r_i^j(\mathcal {A})\right) ^2 + 4a_{ij\ldots j}r_j(\mathcal {A}). \end{aligned}$$

### *Proof*

First, Let $x=(x_1,\ldots ,x_n)^T$ be an Z-eigenvector of $\mathcal {A}$ corresponding to $\rho (\mathcal {A})$, that is,

6 $$\begin{aligned} \mathcal {A}x^{m-1} = \rho (\mathcal {A}) x, \end{aligned}$$

Assume $0< x_t=\mathop {\max }\nolimits _{i \in N} x_i$, then, for any $s\ne t$, by using $x_t^{m-1}\le x_t$, $x_s^{m-1}\le x_s$, we get

7 $$(\rho (\mathcal {A})-a_{t\ldots t}) x_t^{m-1} - a_{ts\ldots s}x_s^{m-1} \le \mathop{\mathop{\sum}\limits_{\delta _{ti_2\ldots i_m}= 0,}}\limits_{\delta _{si_2\ldots i_m}= 0} a_{ti_2\ldots i_m}x_{i_2}\ldots x_{i_m},$$

8 $$(\rho (\mathcal {A})-a_{s\ldots s}) x_s^{m-1} - a_{st\ldots t}x_t^{m-1} \le \mathop{\mathop{\sum}\limits _{\delta _{ti_2\ldots i_m}= 0,}}\limits_{\delta _{si_2\ldots i_m}= 0} a_{si_2\ldots i_m}x_{i_2}\ldots x_{i_m}.$$

From Corollary 4.10 in Chang et al. (2013), we have

$$\begin{aligned} \rho (\mathcal {A})- a_{i\ldots i} \ge 0, \quad i=1,\ldots ,n. \end{aligned}$$

Then, from (7) and (8), we obtain, we obtain

9 $$\begin{aligned} \left( {(\rho (\mathcal {A}) - a_{s \ldots s} )(\rho (\mathcal {A}) - a_{t \ldots t} ) - a_{st \ldots t} a_{ts \ldots s} } \right) x_t^{m - 1}&\le (\rho (\mathcal {A}) - a_{s \ldots s} )\mathop{\mathop{\sum}\limits _{\delta _{ti_2\ldots i_m} = 0,}}\limits_ {\delta _{si_2\ldots i_m}= 0} a_{ti_2\ldots i_m}x_{i_2}\ldots x_{i_m} \nonumber \\&+ a_{ts \ldots s} \mathop{\mathop{\sum}\limits _{\delta _{ti_2\ldots i_m}= 0,}}\limits_ {\delta _{si_2\ldots i_m}= 0} a_{si_2\ldots i_m}x_{i_2}\ldots x_{i_m}. \end{aligned}$$

Recalling that $0< x_t=\mathop {\max}\nolimits _{i \in N} x_i$, we have

10 $$\begin{aligned} (\rho (\mathcal {A}) - a_{s \ldots s} )(\rho (\mathcal {A}) - a_{t \ldots t} ) - a_{st \ldots t} a_{ts \ldots s}&\le (\rho (\mathcal {A}) - a_{s \ldots s} )\mathop{\mathop{\sum}\limits _{\delta _{ti_2\ldots i_m} = 0,}}\limits_{\delta _{si_2\ldots i_m}= 0} a_{ti_2\ldots i_m}\frac{x_{i_2}}{x_t}\ldots \frac{x_{i_m}}{x_t} \nonumber \\&\quad + a_{ts \ldots s} \mathop{\mathop{\sum}\limits _{\delta _{ti_2\ldots i_m}= 0,}}\limits_{\delta _{si_2\ldots i_m}= 0} a_{si_2\ldots i_m}\frac{x_{i_2}}{x_t}\ldots \frac{x_{i_m}}{x_t} \nonumber \\&\le (\rho (\mathcal {A}) - a_{s \ldots s} )r_t^s(\mathcal {A}) + a_{ts \ldots s} r_s^t(\mathcal {A}). \end{aligned}$$

Therefore

$$\begin{aligned} \rho (\mathcal {A}) \le \frac{1}{2}\left\{ a_{t\ldots t}+a_{s\ldots s}+r_t^s(\mathcal {A})+\Theta _{t,s}^{\frac{1}{2}}(\mathcal {A})\right\} . \end{aligned}$$

This must be true for every $s\ne t$, then, we get

$$\begin{aligned} \rho (\mathcal {A}) \le \min \limits _{j\in N,j\ne t} \frac{1}{2}\left\{ a_{t\ldots t}+a_{j\ldots j}+r_t^j(\mathcal {A})+\Theta _{t,j}^{\frac{1}{2}}(\mathcal {A})\right\} . \end{aligned}$$

And this could be true for any $t \in N$, that is

$$\begin{aligned} \rho (\mathcal {A}) \le \max \limits _{i\in N} \min \limits _{j\in N,j\ne i}\frac{1}{2}\left\{ a_{i\ldots i}+a_{j\ldots j}+r_i^j(\mathcal {A})+\Theta _{i,j}^{\frac{1}{2}}(\mathcal {A})\right\} . \end{aligned}$$

Thus, we complete the proof. $\square$

### *Remark 1*

Obviously, we can get

$$\begin{aligned} \max \limits _{i\in N} \min \limits _{j\in N,j\ne i}\frac{1}{2}\left\{ a_{i\ldots i}+a_{j\ldots j}+r_i^j(\mathcal {A})+\Theta _{i,j}^{\frac{1}{2}}(\mathcal {A})\right\} \le \max \limits _{i,j\in N,j\ne i}\frac{1}{2}\left\{ a_{i\ldots i}+a_{j\ldots j}+r_i^j(\mathcal {A})+\Theta _{i,j}^{\frac{1}{2}}(\mathcal {A})\right\} . \end{aligned}$$

That is to say, the bound in Theorem 6 is always better than the result in Theorem 5.

We denote

$$\begin{aligned} \Delta _i= & {} \left\{ {(i_2 ,i_3 , \ldots ,i_m ):i_j = i \quad \text{for} \; \text{some}\ \ j \in \left\{ {2, \ldots ,m} \right\} } \right\} , \quad \text{where}\; i,i_2 , \ldots ,i_m \in N,\\ \overline{\Delta } _i= & {} \left\{ {(i_2 ,i_3 , \ldots ,i_m ):i_j \ne i \quad \text{for}\; \text{any}\ \ j \in \left\{ {2, \ldots ,m} \right\} } \right\} , \quad \text{where}\; i,i_2 , \ldots ,i_m \in N. \end{aligned}$$

And let

$$\begin{aligned} r_i^{\Delta _j } (\mathcal {A}) = \mathop{\mathop{\sum}\limits _{(i_2 , \ldots ,i_m ) \in \Delta _j} ,}\limits_ {\delta _{ii_2 \ldots i_m } = 0 } {\left| {a_{ii_2 \ldots i_m } } \right| }, \quad r_i^{{\overline{\Delta }} _j } (\mathcal {A}) = \sum \limits _{(i_2 , \ldots ,i_m ) \in {\overline{\Delta }} _j } {\left| {a_{ii_2 \ldots i_m } } \right| }. \end{aligned}$$

Then, $$r_i(\mathcal {A})=r_i^{\Delta _j }+r_i^{{\overline{\Delta }} _j } (\mathcal {A})$$.

### **Theorem 7**

*Suppose that* $\mathcal {A}=(a_{i_1i_2 \ldots i_m}) \in {\mathbb{R}}_+^{[m,n]}$ *is an irreducible weakly symmetric tensor. Then*

$$\begin{aligned} \rho (\mathcal {A}) \le \max \limits _{i\in N} \min \limits _{j\in N,j\ne i}\frac{1}{2}\left\{ a_{i\ldots i}+a_{j\ldots j}+r_i^{\overline{\Delta }_j}(\mathcal {A})+\Omega _{i,j}^{\frac{1}{2}}(\mathcal {A})\right\} , \end{aligned}$$

where

$$\begin{aligned} \Omega _{i,j}(\mathcal {A})=\left( a_{i\ldots i}-a_{j\ldots j}+r_i^{\overline{\Delta }_j}(\mathcal {A})\right) ^2 + 4r_i^{\Delta _j}(\mathcal {A})r_j(\mathcal {A}). \end{aligned}$$

### *Proof*

First, Let $x=(x_1,\ldots ,x_n)^T$ be an Z-eigenvector of $\mathcal {A}$ corresponding to $\rho (\mathcal {A})$, that is,

11 $$\begin{aligned} \mathcal {A}x^{m-1} = \rho (\mathcal {A}) x, \end{aligned}$$

Assume $0< x_t=\mathop {\max }\nolimits _{i \in N} x_i$, then, we can get

12 $$\begin{aligned} \rho (\mathcal {A}) x_t&= \sum \limits _{(i_2 , \ldots ,i_m ) \in {\Delta } _s } { {a_{ti_2 \ldots i_m }x_{i_2}\ldots x_{i_m} } }+ \sum \limits _{(i_2 , \ldots ,i_m ) \in {\overline{\Delta }} _s } { {a_{ti_2 \ldots i_m }x_{i_2}\ldots x_{i_m} } } \nonumber \\&\le \sum \limits _{(i_2 , \ldots ,i_m ) \in {\Delta } _s } { {a_{ti_2 \ldots i_m }x_s} }+ \sum \limits _{(i_2 , \ldots ,i_m ) \in {\overline{\Delta }} _s } { {a_{ti_2 \ldots i_m }x_{i_2}\ldots x_{i_m} } }. \end{aligned}$$

That is

13 $$\begin{aligned} (\rho (\mathcal {A})-a_{t\ldots t}) x_t - r_t^{\Delta _s } (\mathcal {A})x_s \le \mathop{\mathop{\sum}\limits _{(i_2 , \ldots ,i_m ) \in {\overline{\Delta }} _s,}}\limits_{\delta _{ti_2\ldots i_m}= 0} a_{ti_2\ldots i_m}x_{i_2}\ldots x_{i_m}, \end{aligned}$$

Similarly, we can get

14 $$\begin{aligned} (\rho (\mathcal {A})-a_{s\ldots s}) x_s - r_s^{{\Delta } _t } (\mathcal {A})x_t \le \sum \limits _{(i_2 , \ldots ,i_m ) \in {\overline{\Delta }} _t } { {a_{si_2 \ldots i_m }x_{i_2}\ldots x_{i_m} } }. \end{aligned}$$

From Corollary 4.10 in Chang et al. (2013), we have

$$\begin{aligned} \rho (\mathcal {A})- a_{i\ldots i} \ge 0, \quad i=1,\ldots ,n. \end{aligned}$$

Then, from (13) and (14), we obtain, we obtain

15 $$\begin{aligned} \left( {(\rho (\mathcal {A}) - a_{s \ldots s} )(\rho (\mathcal {A}) - a_{t \ldots t} ) - r_t^{\Delta _s} (\mathcal {A}) r_s^{{\Delta } _t } (\mathcal {A}) } \right) x_t&\le (\rho (\mathcal {A}) - a_{s \ldots s} )\mathop{\mathop{\sum}\limits _{(i_2 , \ldots ,i_m ) \in {\overline{\Delta }} _s,}}\limits_ {\delta _{ti_2\ldots i_m}= 0} a_{ti_2\ldots i_m}x_{i_2}\ldots x_{i_m} \nonumber \\&\quad + r_t^{\Delta _s} (\mathcal {A}) \sum \limits _{(i_2 , \ldots ,i_m ) \in {\overline{\Delta }} _t } { {a_{si_2 \ldots i_m }x_{i_2}\ldots x_{i_m} } }. \end{aligned}$$

Recalling that $0< x_t=\mathop {\max }\nolimits _{i \in N} x_i$, we have

16 $$\begin{aligned} (\rho (\mathcal {A}) - a_{s \ldots s} )(\rho (\mathcal {A}) - a_{t \ldots t} ) - r_t^{\Delta _s} (\mathcal {A}) r_s^{{\Delta } _t } (\mathcal {A}) \le (\rho (\mathcal {A}) - a_{s \ldots s} )r_t^{{\overline{\Delta }} _s } (\mathcal {A}) + r_t^{\Delta _s} (\mathcal {A}) r_s^{{\overline{\Delta }} _t } (\mathcal {A}). \end{aligned}$$

Therefore

$$\begin{aligned} \rho (\mathcal {A}) \le \frac{1}{2}\left\{ a_{t\ldots t}+a_{s\ldots s}+r_t^{\overline{\Delta }_s}(\mathcal {A})+\Omega _{t,s}^{\frac{1}{2}}(\mathcal {A})\right\} . \end{aligned}$$

This must be true for every $s\ne t$, then, we get

$$\begin{aligned} \rho (\mathcal {A}) \le \min \limits _{j\in N,j\ne t} \frac{1}{2}\left\{ a_{t\ldots t}+a_{j\ldots j}+r_t^{\overline{\Delta }_s}(\mathcal {A})+\Omega _{t,j}^{\frac{1}{2}}(\mathcal {A})\right\} . \end{aligned}$$

And this could be true for any $t \in N$, that is

$$\begin{aligned} \rho (\mathcal {A}) \le \max \limits _{i\in N} \min \limits _{j\in N,j\ne i}\frac{1}{2}\left\{ a_{i\ldots i}+a_{j\ldots j}+r_i^{\overline{\Delta }_j}(\mathcal {A})+\Omega _{i,j}^{\frac{1}{2}}(\mathcal {A})\right\} . \end{aligned}$$

Thus, we complete the proof. $\square$

### *Remark 2*

Let $\Pi _i$ be a nonempty proper subset of $\Delta _i$, we have that for $(i_2, \ldots , i_m) \in \Pi _i$,

$$\begin{aligned} r_i^{\Delta _i}(\mathcal {A})=r_i^{\Pi _i}(\mathcal {A})+r_i^{\overline{\Pi }_i}(\mathcal {A}). \end{aligned}$$

Similar to the proof of Theorem 7, we can get

$$\begin{aligned} \rho (\mathcal {A}) \le \min \limits _{\Pi _i \in \Delta _i} \max \limits _{i\in N} \min \limits _{j\in N,j\ne i}\frac{1}{2}\left\{ a_{i\ldots i}+a_{j\ldots j}+r_i^{\overline{\Pi }_j}(\mathcal {A})+\Upsilon _{i,j}^{\frac{1}{2}}(\mathcal {A})\right\} , \end{aligned}$$

where

$$\begin{aligned} \Upsilon _{i,j}(\mathcal {A})=\left( a_{i\ldots i}-a_{j\ldots j}+r_i^{\overline{\Pi }_j}(\mathcal {A})\right) ^2 + 4r_i^{\Pi _j}(\mathcal {A})r_j(\mathcal {A}), \end{aligned}$$

which is always better than the result in Theorem 6.

### *Example 1*

We now show the efficiency of the new upper bounds in Theorems 6 and 7 by the following example. Consider the tensor $\mathcal {A}=(a_{ijk})$ and of order 3 dimension 3 with entries defined as follows:

$$\begin{aligned} a_{111}=\frac{1}{2}, \quad a_{222}=1, \quad a_{333}=3, \quad \text{ and } \ \ a_{ijk}=\frac{1}{3} \quad \text{ elsewhere}. \end{aligned}$$

By Theorem 1, we have

$$\begin{aligned} \rho (\mathcal {A}) \le 9.8150. \end{aligned}$$

By Theorem 2, we have

$$\begin{aligned} \rho (\mathcal {A}) \le 5.6667. \end{aligned}$$

By Theorem 3, we have

$$\begin{aligned} \rho (\mathcal {A}) \le 5.6079. \end{aligned}$$

By Theorem 4, we have

$$\begin{aligned} \rho (\mathcal {A}) \le 5.5494. \end{aligned}$$

By Theorem 5, we have

$$\begin{aligned} \rho (\mathcal {A}) \le 5.5296. \end{aligned}$$

By Theorem 6, we have

$$\begin{aligned} \rho (\mathcal {A}) \le 5.5107. \end{aligned}$$

By Theorem 7, we have

$$\begin{aligned} \rho (\mathcal {A}) \le 5.3654. \end{aligned}$$

This example shows that the bound in Theorem 7 is the best among the known bounds.

## Conclusion

In this paper, we presented some bounds for the largest Z-eigenvalue of an irreducible weakly symmetric and nonnegative tensor. These bounds are always sharper than the bounds in Chang et al. (2013), Song and Qi (2013), He and Huang (2014), Li et al. (2015), He (2016).
